# Telomere length and telomerase activity in non-small cell lung cancer prognosis: clinical usefulness of a specific telomere status

**DOI:** 10.1186/s13046-015-0195-9

**Published:** 2015-08-07

**Authors:** Tamara Fernández-Marcelo, Ana Gómez, Irene Pascua, Carmen de Juan, Jacqueline Head, Florentino Hernando, Jose-Ramón Jarabo, Joaquín Calatayud, Antonio-José Torres-García, Pilar Iniesta

**Affiliations:** Department of Biochemistry and Molecular Biology II. Faculty of Pharmacy, Complutense University, Sq. Ramón y Cajal s/n (Ciudad Universitaria), Madrid, 28040 Spain; Service of Thoracic Surgery, San Carlos Hospital, Madrid, 28040 Spain; Service of General Surgery and Digestive Tract, San Carlos Hospital, Madrid, 28040 Spain; Sanitary Research Institute of San Carlos Hospital (IdISSC), Madrid, 28040 Spain

**Keywords:** Non-small cell lung cancer, Prognosis, Telomerase, Telomeres

## Abstract

**Background:**

Considering previous data and the need to incorporate new biomarkers for the prognosis of solid tumours into the clinic, our aim in this work consists of evaluating the potential clinical use of telomeres and telomerase in non-small cell lung cancer (NSCLC).

**Methods:**

Telomere status was established by determination of telomere length using the Terminal Restriction Fragment length method, and telomerase activity by the Telomeric Repeat Amplification Protocol in 142 NSCLCs and their corresponding control samples, obtained from patients submitted to surgery. Group-oriented curves for disease-free survival were calculated according to the Kaplan-Meier method considering telomere length, T/N ratio (telomere length in tumour to control tissue) and telomerase activity.

**Results:**

Overall, tumours had significantly shorter telomeres compared with telomeres in control tissues (*P* = 0.027). More than 80 % of NSCLCs displayed telomerase activity. Regarding prognosis studies, patients whose tumours showed a mean telomere length (MTL) <7.29 Kb or T/N ratio <0.97 showed a significantly poor clinical evolution (*P* = 0.034 and *P* = 0.040, respectively). As result of a Cox multivariate analysis including pathologic state and lymph node dissemination, the MTL and T/N ratio emerged as independent significant prognostic factors.

**Conclusions:**

Telomerase activity was identified as a marker of poor prognosis. The novel finding of this study is the independent prognosis role of a specific telomere status in NSCLC patients. According to our results, telomere function may emerge as a useful molecular tool that allow to select groups of NSCLC patients with different clinical evolution, in order to establish personalized therapy protocols.

## Background

Lung cancer remains the most common cancer in men worldwide [1] and the identification of subjects at higher risk of cancer progression and recurrence, who would benefit from more specific therapies, is necessary [2]. The risk assessment by the Tumour-Nodes-Metastasis (TNM) staging system is relatively inexact as it only considers tumour characteristics at a particular moment (TNM at diagnosis) [3]. Of fundamental concern is the identification and use of new biomarkers, with high specificity and sensitivity, which will allow the prediction of the disease course and identification of patients who would or would not benefit from specific therapy. In this way, telomere function has been recognized as possible biomarker [2]. However, it has not yet been established a consensus for the use of telomere function in cancer clinical practice.

Telomeres are ribonucleoprotein complexes located at the ends of eukaryotic chromosomes which consist of tandem repeats of a DNA sequence (TTAGGG in all vertebrates) [4]. Among its main functions, telomeres mask double strand break DNA damage signals at the extreme of chromosome and prevent chromosomal fusions [5–8]. In the context of a somatic cell, a significant telomere shortening is monitored by p53 and RB1 and leads to cell death or senescence. However, cells that surpass their normal replicative limit and continue to divide lose all remaining protective telomeric DNA and enter the crisis stage, characterised by massive genomic instability and cell death [9]. At this point, transformed clones emerge and telomerase activation is detectable in the majority of tumour cells and is the main positive power for telomere preservation and elongation [10].

Telomeres and telomerase play a crucial role in human carcinogenesis: the majority of tumours have telomere length alterations which can eventually lead to telomere dysfunction [11]. A disruption of telomere length homeostasis affects telomere structure and leads to genomic instability by generating chromosome end-to end fusions and chromosomal abnormalities [5]. In relation to telomerase, an increase in telomerase activity is often directly correlated with uncontrolled growth of cells, a known hallmark of cancer, and has become a widely acceptable tumour marker and a popular target for anticancer therapeutics [12].

Considering previous data and the need to incorporate new biomarkers for the prognosis of solid tumours into the clinic, our first aim in this work consists of evaluating the potential clinical use of telomeres and telomerase in NSCLC. With this objective, we analyzed both parameters directly implicated in telomere function in a large series of cases of NSCLC. Thus, we identified groups of NSCLC patients with different clinical evolution, independently of the TNM stage tumours, in relation to telomere status (telomere length & T/N ratio).

## Methods

### Patients and tissue samples

One hundred and forty-two primary NSCLCs were obtained from patients who had undergone potentially curative surgery at San Carlos Hospital in Madrid, Spain. Paired non-tumour tissues from the same patient, used as controls, were obtained and microscopically confirmed. After surgical resection, all tissue samples were snap-frozen in liquid nitrogen and stored at −80 °C until processed. Cryostat sectioned, H&E stained samples from each tumour block were examined microscopically by two independent pathologists to confirm the presence of ≥80 % tumour cells. All the cases were collected without selection in function of gender, age or tumour stage and no patient had received previous chemotherapy or radiotherapy before diagnosis and entry into this study. Informed consent was obtained from patients prior to investigation and this study was approved by the Ethical Committee of the Hospital.

NSCLCs were staged pathologically using the TNM system 6^th^ Edition [13] and consisted of 86 TNM I tumours, 9 TNM II tumours, 38 TNM IIIA tumours, 5 TNM IIIB tumours and 4 TNM IV tumours. The median follow-up period of patients was 5 years (range, 1–133 months).

### Telomere length measurement

Terminal Restriction Fragment (TRF) length measurement was performed using Telo TTAGGG Telomere Length Assay kit (Roche Applied Science, Germany) as previously described [14]. TRF lengths for tumour and control tissues were determined by comparing the signals relative to a standard molecular weight using Image Gauge software (version 3.46; Fujifilm, Japan). The TRF length ratio was determined as the ratio of the length of tumour tissue TRF and their paired normal tissue TRF (T/N ratio).

### Telomerase activity determination

Telomerase activity was measured using the Telomeric Repeat Amplification protocol (TRAP)-based telomerase polymerase chain reaction (PCR)-enzyme-linked immunosorbent assay (ELISA) kit (Roche Applied Science, Germany), as described earlier [15]. Considering that the cut-off for TRAP-ELISA negativity corresponds to an optical density (OD) _450 nm_ < 0.2, all samples with OD _450 nm_ ≥ 0.2 were considered telomerase positive [15].

### Statistical analysis

Statistical analyses were performed using the SPSS software package (version 19.0 SPSS, Chicago, IL, USA). Differences in telomere length and T/N ratio among various groups of patients, discriminated for clinical variables, were analysed by the Student-T and ANOVA tests, or their non-parametric alternatives, Mann-Whitney U test and Kruskal-Wallis test. *P*-value < 0.05 was considered statistically significant. The paired samples T test was used for comparing the means of two related variables.

Group-oriented curves for disease-free survival (DFS) were calculated according to the Kaplan-Meier method considering telomere length, T/N ratio and telomerase activity. DFS was calculated from the day of surgery until recurrence. The differences in DFS across different groups were compared using the log-rank test. The relative prognostic impact of telomere length, T/N ratio and telomerase activity, compared with established prognosis factors, was analysed using Cox multivariate analysis.

Cutoff Finder Web Application [16] was used to determine the cut-off points for prognosis analysis.

## Results

### Telomere status and telomerase activity in tissue samples

We evaluated telomere length in a total of 284 lung tissue samples: 142 NSCLCs and the corresponding non-tumour samples. The mean telomere length (mean ± standard error) was 6.56 ± 0.26 Kb in NSCLCs and 7.00 ± 0.19 Kb for non-tumour samples. Overall, tumours had significantly shorter telomeres than matched non-tumour tissues (*P* = 0.027; paired T test). The T/N ratio was 0.93 ± 0.026. Positive results for telomerase activity were found in 123 (86.6 %) of 142 NSCLCs; 19 tumours (13.4 %) were telomerase negative.

### Telomere status and telomerase activity: correlation with clinical variables of non-small cell lung tumours

As it is showed in the Table 1, there was a statistically significant association between the telomere length and the size of the primary tumour (T), (*P* = 0.006; Kruskal-Wallis test): T1 tumours had shorter telomeres than both T2 and T3 tumours. T4 tumours, comprised of a limited number of cases, had a mean telomere length close to T1 tumours. Moreover, there were significant differences between the T/N ratio and T descriptor (*P* = 0.024; Kruskal-Wallis test). A significant association was found between the tumour histology and the T/N ratio (*P* = 0.022; Kruskal-Wallis test), which was significantly diminished in large cell undifferentiated carcinomas compared to squamous cell carcinomas and adenocarcinomas. No association was found neither to telomere length nor T/N ratio for the gender, TNM stage, lymph node dissemination (N) and tumour metastasis (M) in NSCLCs (Table 1).

**Table 1:** Telomere status and clinical variables in non-small cell lung cancers

Variable	N° of cases	Telomere length	P and test statistic	T/N ratio	P and test statistic
		(Kilobase pairs; mean ± standard error)		(mean ± standard error)	
Gender	142				
Female	12	5.49 ± 0.69	0.274; Mann-Whitney U Test	0.83 ± 0.07	0.416; Mann-Whitney U Test
Male	130	6.66 ± 0.28		0.94 ± 0.03	
TNM stage	142				
I	86	6.08 ± 0.29	0.204; one-way ANOVA	0.91 ± 0.03	0.442; Kruskal-Wallis test
II	9	7.01 ± 1.37		0.96 ± 0.11	
IIIA	38	7.45 ± 0.57		1.00 ± 0.06	
IIIB	5	6.15 ± 1.55		0.85 ± 0.18	
IV	4	7.79 ± 2.84		0.80 ± 0.10	
Size of the primary tumour, T	142				
T1	22	5.54 ± 0.52	0.006; Kruskal-Wallis test	0.93 ± 0.04	0.024; Kruskal-Wallis test
T2	91	6.32 ± 0.30		0.90 ± 0.03	
T3	22	8.84 ± 0.84		1.11 ± 0.09	
T4	7	5.75 ± 1.10		0.79 ± 0.13	
Lymph node dissemination, N	142				
N0	102	6.44 ± 0.30	0.532; Kruskal-Wallis test	0.93 ± 0.03	0.456; Kruskal-Wallis test
N1	11	7.74 ± 1.22		1.02 ± 0.10	
N2	28	6.64 ± 0.62		0.93 ± 0.07	
N3	1	3.25		0.62	
Tumour metastasis, M	142				
Absence, M0	138	6.52 ± 0.26	0.848; Mann-Whitney U Test	0.94 ± 0.03	0.349; Mann-Whitney U Test
Presence, M1	4	7.79 ± 2.84		0.80 ± 0.10	
Histology	141^a^				
Squamous cell carcinoma (SCC)	79	6.47 ± 0.33	0.128; Kruskal-Wallis test	0.94 ± 0.03	0.022; Kruskal-Wallis test
Adenocarcinoma (AD)	56	6.93 ± 0.46		0.96 ± 0.04	
Large cell undifferentiated carcinoma (LCUC)	6	4.31 ± 0.89		0.64 ± 0.10	

^a^Missing data

Finally, applying a Chi-square test a statistical association between telomerase activity and the gender (*P* = 0.034), TNM stage (*P* = 0.042), tumour metastasis (*P* = 0.029) and tumour histology (*P* = 0.019) was found (Table 2).

**Table 2:** Telomerase activity and clinical variables in non-small cell lung cancers

Variable	N° of cases	Telomerase activity		P; Chi-square test
		Negative	Positive	
Gender	142			
Female	12	4	8	0.034
Male	130	15	115	
TNM stage	142			
I	86	12	74	0.042
II	9	1	8	
IIIA	38	2	36	
IIIB	5	2	3	
IV	4	2	2	
Size of the primary tumour, T	142			
T1	22	3	19	0.126
T2	91	11	80	
T3	22	2	20	
T4	7	3	4	
Lymph node dissemination, N	142			
N0	102	13	89	0.838
N1	11	1	10	
N2	28	5	23	
N3	1	0	1	
Tumour metastasis, M	142			
Absence, M0	138	17	121	0.029
Presence, M1	4	2	2	
Histology	141^a^			
Squamous cell carcinoma (SCC)	79	8	71	0.019
Adenocarcinoma (AD)	56	7	49	
Large cell undifferentiated carcinoma (LCUC)	6	3	3	

^a^Missing data

Considering our studied population, no significant differences were found between the telomere lengths of negative and positive telomerase activity tumours (*P* = 0.549; Mann-Whitney U test). The mean telomere length (mean ± typical error) was 7.16 ± 0.87 Kb in negative telomerase activity tumours and 6.47 ± 0.27 Kb, for positive telomerase activity tumours. The range of tumour telomere lengths between the groups defined by telomerase activity was similar: 1.51–16.49 Kb for negative telomerase activity tumours and 1.42–17.50 Kb for positive telomerase activity tumours.

### Telomere status, telomerase activity and NSCLC patients prognosis

**Fig. 1:**
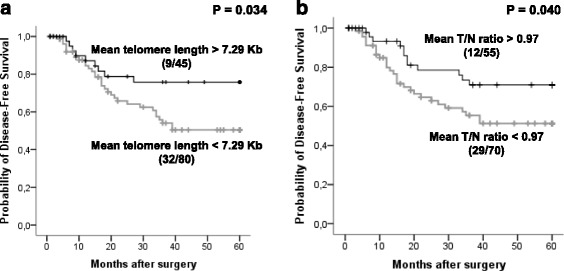
Kaplan-Meier plots of Disease Free Survival (DFS) considering telomere status. Kaplan-Meier survival curves in relation to telomere length (**a**) and T/N ratio (**b**) in non-small cell lung cancer. Numbers in brackets represent cases with tumour recurrence and crosses indicate censored data

Prognosis studies were developed to assess the impact on clinical course of telomere status (telomere length & T/N ratio) and telomerase activity in patients with resected NSCLCs. Only patients who had undergone potentially curative surgery and who did not die during the post-operatory period were considered in these studies, as established in the literature. A total of 125 cases were considered. First, patients were stratified into two groups according to the mean telomere length using the Cutoff Finder Web Application [16]: the group of patients who had tumours with a mean telomere length <7.29 Kb presented a significantly poor clinical evolution, compared to the group whose mean tumour telomere length was >7.29 Kb (*P* = 0.034; Log-Rank. Fig. 1a). Forty percent (32/80) of patients included in the first group relapsed during the follow-up period, compared with the second group where 20 % of patients (9/45) experienced a relapse. The Kaplan-Meier curves show this relationship between the telomere length and disease-free survival (Fig. 1a).

Next, using the Cutoff Finder Web Application [16] the best prognostic cut-off for the T/N ratio was set in 0.97: patients who had tumours with a T/N ratio <0.97 had a significantly shorter disease-free survival compared with patients who had a bigger T/N ratio (*P* = 0.040; Log-Rank. Fig. 1b).

As result of a Cox multivariate analysis, including TNM stage and lymph node dissemination (only these factors had prognostic relevance in the univariate analysis, *P* < 0.001), telomere length emerged as an independent significant prognostic factor (Table 3A and B). In both cases, the patients with a mean telomere length <7.29 Kb were at increased risk for recurrence. Also using the Cox multivariate analysis, a mean T/N ratio <0.97 was found to be a prognostic factor independent of the TNM stage (*P* = 0.041) (Table 4A and B).

**Table 3:** Multivariate Cox Regression Analysis considering TNM stage (A), lymph node dissemination (B) and telomere length for 125 patients with non-small cell lung cancer

**(A) Variable**	**HR (95 % CI)**	**P**
Mean telomere length in NSCLCs <7.29 Kb	2.70 (1.27–5.76)	0.010
TNM stage, I *vs.* II or IIIA	0.34 (0.17–0.67)	0.002
**(B) Variable**	**RR (95 % CI)**	**P**
Mean telomere length in NSCLCs <7.29 Kb	2.26 (1.07–4.76)	0.032
Lymph node dissemination (N), negative (N0) *vs*. positive (N1 or N2)	0.41 (0.19–0.88)	0.021

*HR* hazard ratio, *CI* confidence interval

**Table 4:** Multivariate Cox Regression Analysis considering TNM stage (A), lymph node dissemination (B) and T/N ratio for 125 patients with non-small cell lung cancer

**(A) Variable**	**HR (95 % CI)**	**P**
Mean T/N ratio <0.97	2.02 (1.03–3.97)	0.041
TNM stage, I *vs*. II or IIIA	0.42 (0.21–0.81)	0.010
**(B) Variable**	**RR (95 % CI)**	**P**
Mean T/N ratio <0.97	1.93 (0.98–3.81)	0.058
Lymph node dissemination (N), negative (N0) *vs*. positive (N1 or N2)	0.44 (0.21–0.95)	0.036

*HR* hazard ratio, *CI* confidence interval

**Fig. 2:**
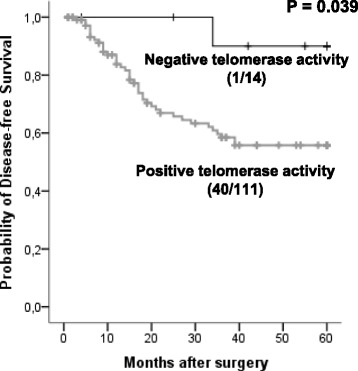
Kaplan-Meier plots of Disease Free Survival (DFS) considering telomerase activity. Kaplan-Meier survival curves in relation to telomerase activity. Numbers in brackets represent cases with tumour recurrence and crosses indicate censored data

Finally, telomerase activity discriminated between two groups of patients: the absence of telomerase activity in the tumour conferred a better clinical evolution (*P* = 0.039; Log-Rank. Fig. 2). In multivariate analysis, a clear trend towards a potential role for telomerase activity as an independent prognostic factor of the TNM stage (*P* = 0.089) and lymph node dissemination (*P* = 0.101) was found.

## Discussion

Malignant cells, in general, have shorter telomeres than their normal counterparts [17] as a reflection of their extended proliferation. In fact, diseases of high cellular turnover are associated with telomere shortening, telomere dysfunction and cancer predisposition [18]. In bronchial carcinogenesis, telomere shortening is an early event [19] and previous results [20] including those of our research group [15] show a significant telomere shortening in NSCLCs in relation to the control tissues. In other tumour types, telomere attrition has been also demonstrated [21]. Therefore, the results shown in this paper, as with the previous data, reflect differences between the mean telomere length in NSCLCs and the non-tumour tissues, which are validated by a T/N ratio <1.

Progressive telomere erosion is countered with telomerase, which is expressed in the vast majority of human cancers [12]. Our data indicated more than 80 % of NSCLCs analysed expressed telomerase activity, in agreement with previously published works [15], without significant differences in the mean telomere length values between telomerase positive and negative tumours. Although telomerase activation can be an early event in cancer, it is not necessary for cancer initiation. However, telomerase can stimulate tumour progression by ensuring maintenance of telomeres above a critically short length, thus preventing induction of cellular senescence or apoptosis [22]. Appart from telomerase, possible implication of Alternative Lengthening of Telomeres (ALT) mechanisms in telomere elongation of telomerase-negative tumours should be considered. However, the ALT phenotype was not investigated in our research work considering the limited role of this mechanism in lung cancer. In fact, the ALT phenotype is common in tumours such as osteosarcomas, undifferentiated pleomorphic sarcomas, leiomyosarcomas, astrocytic tumours grades 2 and 3, and pancreatic neuroendocrine tumours [11, 23]. However, ALT is a very infrequent mechanism in the most common cancer types, carcinomas, which are derived from epithelia [24]. Heaphy et al. assessed the ALT phenotype in 6110 primary tumours from 94 different cancer subtypes and this mechanism was not detected in most lung carcinoma subtypes, only individual cases of small cell (2 %) and large cell lung carcinomas (3 %) were observed. ALT-positive cells are characterized by striking telomere length heterogeneity [11]. In the present work, it is noteworthy that the maximum value in telomere length for telomerase positive and negative lung tumours is close to that defined in the normal human population: the length of telomeres is heterogeneous, ranging between 5 and 15 Kb [25]. Therefore, very long telomeres, other feature of ALT, were not detected.

The role of telomeres in the initiation and progression of carcinogenesis has been widely recognized [11]; thus, its relation with clinical variables, in conjunction with others molecular markers, could represent a therapeutic opportunity for cancer patients. Currently, except for recent developments in relation to the involvement of *EGFR* mutational status [26], limited advances in the detection of molecular targets in lung cancer were obtained. For NSCLCs, the statistical association between the tumour size (T descriptor) and telomere status is in line with previously published data [15]: the highest degree of telomere shortening is detected for tumours that grow into the area of mediastinum or cancers in which a malignant pleural effusion is reached (T4).

An altered telomere length in cancer cells could give the ability to metastasize and cause recurrent disease and, in consequence, be a predictor of clinical outcome. Previous works concluded that careful assessment of telomere length or its proxies, such as DNA content, will be part of novel risk assessment and prognostic modalities for patients [2]. More recently, in glioblastoma multiform tumours, telomeres were always shorter when compared with normal brain tissue, and together with telomerase activity seem to be associated with malignancy and poor outcome [27]. In lung cancer, previous studies have evaluated whether telomere length could represent a risk factor or a prognostic marker, however most of them are contradictory: for NSCLCs patients both shorter and longer telomeres has been associated with decreased overall survival [28]. Results obtained in the present work indicate that the worst prognosis is seen in patients whose mean tumour telomere length is lower than 7.29 Kb or when almost any degree of telomere shortening is reached in tumours cells. Both parameters related to telomere status proved to be independent of other known prognostic factors. However, we also demonstrated in colorectal cancer that telomere attrition conferred good clinical evolution. Therefore, cancer prognosis associated to telomere status could be dependent on the tumour type [14]. A deficiency in the function of senescence and cell death pathways [29], or other molecules related to telomere maintenance and genome instability [30] could explain the unfavorable prognosis for post-surgical patients with NSCLCs and short telomeres. Genetic context underlying telomere status, which confers the different clinical outcome, must be carefully considered.

Recently, two works have demonstrated that leukocyte relative telomere length in peripheral blood is an independent prognostic marker in glioma and gastric cancer patients, being shorter telomeres associated with the worst clinical evolution of patients [31, 32]. Telomere analyses in leukocytes from peripheral blood could be a useful biomarker to improve the prognosis prediction in cancer patients. However, previously it is necessary corroborate and validate correlations between telomere status in blood cells and tumours through prospective studies. This is a field that needs to be investigated in lung cancer.

Finally, the presence of telomerase activity in NSCLCs conferred the worst outcome, confirming previous results [15]. Telomerase activity or hTERT expression has proved to be a marker of malignancy. Furthermore, its diagnostic utility is being demonstrated [11].

## Conclusions

The novel finding of this study is the independent prognosis role of a specific telomere status in NSCLC patients. Moreover, telomerase activity is confirmed as a prognostic marker in NSCLC. According to results from the present work, telomere function may emerge as a useful molecular tool that allow to select groups of NSCLC patients with different clinical evolution, in order to establish personalized therapy protocols.
